# RNA-Seq Analysis Reveals Hub Genes Involved in Chicken Intramuscular Fat and Abdominal Fat Deposition During Development

**DOI:** 10.3389/fgene.2020.01009

**Published:** 2020-08-28

**Authors:** Siyuan Xing, Ranran Liu, Guiping Zhao, Lu Liu, Martien A. M. Groenen, Ole Madsen, Maiqing Zheng, Xinting Yang, Richard P. M. A. Crooijmans, Jie Wen

**Affiliations:** ^1^State Key Laboratory of Animal Nutrition, Key Laboratory of Animal (Poultry) Genetics Breeding and Reproduction, Ministry of Agriculture and Rural Affairs, Institute of Animal Sciences, Chinese Academy of Agricultural Sciences, Beijing, China; ^2^Animal Breeding and Genomics, Wageningen University and Research, Wageningen, Netherlands

**Keywords:** chicken, intramuscular fat, abdominal fat, transcriptome, tissue development

## Abstract

Fat traits are important in the chicken industry where there is a desire for high intramuscular fat (IMF) and low abdominal fat. However, there is limited knowledge on the relationship between the dynamic status of gene expression and the body fat deposition in chicken. Transcriptome data were obtained from breast muscle and abdominal fat of female chickens from nine developmental stages (from embryonic day 12 to hatched day 180). In total, 8,545 genes in breast muscle and 6,824 genes in abdominal fat were identified as developmentally dynamic genes. Weighted correlation network analysis was used to identify gene modules and the hub genes. Twenty-one hub genes were identified, e.g., *ENSGALG00000041996*, which represents a candidate for high IMF, and *CREB3L1*, which relates to low abdominal fat weight. The transcript factor *L3MBTL1* and the transcript factor cofactors *TNIP1*, *HAT1*, and *BEND6* related to both high breast muscle IMF and low abdominal fat weight. Our results provide a resource of developmental transcriptome profiles in chicken breast muscle and abdominal fat. The candidate genes can be used in the selection for increased IMF content and/or a decrease in abdominal fat weight which would contribute to the improvement of these traits.

## Introduction

Lipid metabolism, regulation, and deposition play a very important role not only in relation to obesity in humans but also in livestock production because of its close relationship with tasty and healthy food supply for humans. For global meat consumption, chicken meat is the second largest, providing one fourth of meat resource^1^. In the Chinese meat-type chicken industry, yellow-feathered dwarf chickens are used in one third of the breeding system. High intramuscular fat (IMF) content contributes to high meat quality, and as a result, increasing IMF deposition is a desirable goal in meat-type chicken breeding. Genetic selection, nutritional strategies, and management practices have been shown to enhance fat deposition and IMF in swine (Reiter et al., 2007) and cattle (Pethick et al., 2001). However, unlike the muscle type of pork and beef, the marbling in chicken meat is almost invisible. In chicken, an increased IMF in muscle tissue will result in an increase in abdominal fat (AF) deposition in the chicken body (Jiang et al., 2017). Excess of AF influences animal welfare and becomes a waste product for human consumption after slaughter, therefore resulting in considerable economic losses (Jiang et al., 2017). Thus, an increase of IMF and a reduction of AF deposition are important goals of meat-type chicken production.

Fat can be deposited at different sites in the chicken body: around abdominal tissues (AF, also called visceral fat or central fat), in bones (marrowfat), under the skin (subcutaneous fat), and in the muscle (IMF). The IMF content plays a key role in various quality traits of meat, and it varies between different chicken breeds/lines and tissue types and also varies with age, gender, feeding, and even during the season (Hocquette et al., 2010). AF is the most dominant fat tissue in the mature animal body. Fat tissue is composed of adipocytes, which mainly differentiate from mesenchymal stem cells (MSCs) (Pittenger et al., 1999). Adipocyte differentiation should be characterized by two phases, the determination phase (hyperplasia) and the terminal differentiation phase (hypertrophy) (Symonds, 2012). Although it has been suggested that the number of adipocytes will not increase after adulthood, in humans, prolonged obesity in adults can result in an increased number of adipocytes (Schmitz et al., 2016). For meat-type animals, at the cellular level, the adipocyte number increases most rapidly in the abdominal wall and minimally in the intramuscular depot (Allen, 1976). Chicken fat deposition varies during the different developmental stages. In embryonic stages, the fat deposition starts in the muscle (IMF) before deposition around the abdomen, while at the fast-growing stage, it is the other way around.

The gain in fat depends on the adipocyte’s ability to synthesize and store lipids. The molecular mechanisms’ underlying fat deposition and its regulation are still insufficiently understood, but there is a close relationship between adipocyte development and expression of specific genes in pre-adipocytes. This involves genes related to adipocyte differentiation, transcription regulators, and genes related to lipid metabolism (Ono et al., 1990). The transcription factors (TFs) *PPAR*γ, *C/EBPs*, and *ADD1* (*SREBP1*) are involved in the regulation of adipocyte differentiation. *PPAR*γ was shown to be a necessary regulator of induced differentiation of adipocytes (Kim et al., 2014). *C/EBP*α plays a very important role in adipocyte differentiation (Tang and Lane, 1999) and activates genes such as *aP2*, *PEPCK*, and *SCD1*, which all contain TF binding sites for *C/EBP*α (Farmer, 2006).

RNA sequencing has been used in studies of chicken pre-adipocyte development at the cellular level (Guo et al., 2018), chicken fat deposition *in vivo* (Zhuo et al., 2015), and embryonic adipocyte development (Na et al., 2018). Adipogenesis has been shown to be a multistep process, regulated by both enhancers and inhibitors (Tontonoz et al., 1994; Ross et al., 2000). Several tissues are involved in the regulation of fat deposition, and the contribution of these tissues changes during development. Previous studies have mainly focused on IMF and AF separately or mainly focused on one or two developmental stages (Resnyk et al., 2017). Therefore, this study focuses on multiple time points of development and the transcriptome dynamic changes of two different fat-related tissues to achieve more completable knowledge on the molecular mechanism of fat deposition in chicken.

## Materials and Methods

### Animal Genetic Background, Phenotypes, and Sample Collection

The parental generation used in this study was selected from an inbred dwarf yellow-feathered Jingxing-Huang IMF-up selected chicken line, which is a widely used Chinese local meat-type chicken line (Jiang et al., 2017). Twenty roosters and 60 hens (one male mated to three females) were selected to produce the animals of the experimental generation for the phenotype recording and sample collection. In the experimental generation, two batches of eggs were incubated. Sample collection was subsequently performed at the following nine developmental stages: E12 (embryonic day 12), E17, D01 (day 1 after hatching), D07, D21, D56, D98, D140, and D180. Chickens were reared with *ad libitum* access to feed and water. Tissue sampling of the animals was approved by the animal ethics committee of the Institute of Animal Sciences, Beijing, China. The following phenotypes were recorded: body weight, breast muscle weight (BMW), and AF weight (AFW). The organ growth curves of breast muscle (BM) and AF were fitted by the logistic model using the Origin software. BM and AF samples were collected from every animal and developmental stage except for AF from stages E12 to D01, where no obvious AF tissue is observed. From developmental stages E12 to D21, hematoxylin–eosin (HE) and Red Oil O stain were used on the BM samples, and the relative amount of BM-IMF during these phases was measured by the Red Oil-stained section. From D21 to D180, the IMF content in BM of the chickens was determined by the Soxhlet extraction method (Soxhlet, 1879). The relative BM-IMF content from E12 to D07 was calculated by the IPP software from 10 captured images of Red Oil O-stained sections. The genders of the embryo were determined by a length polymorphism in the intron of the *CHD1* gene by performing a PCR and analysis of the fragments using agarose gel electrophoresis (Griffiths and Korn, 1997). The sequences of the primers are as follows: forward primer 5′-GTTACTGATTCGTCTACGAGA-3′ and reverse primer 5′-ATTGAAATGATCCAGTGCTTG-3′. Finally, three full-sib families were used as experimental chickens. Each full-sib family provided one chicken for samples for RNA-Seq in each stage. The middle of AF and the pectoralis major of BM samples from 27 female chickens were used for RNA extraction (Table 1). Additionally, in the embryonic period, it is not possible to divide the pectoralis major and pectoralis minor; therefore, the whole BM was used for RNA isolation.

**TABLE 1:** RNA-sequenced sample number of BM and AF in different developmental stages.

Development stage	Breast muscle sample number	Abdominal fat sample number
E12	3	–*
E17	3	–
D1	3	–
D7	3	3
D21	3	3
D56	3	3
D98	3	3
D140	3	3
D180	3	3

*There is no dissectible abdominal fat in E12, E17, and D01.

### RNA Sequencing and Data Quality Control

The QIAGEN RNeasy Kit was used to isolate total RNA, and genomic DNA was removed by using the TIANGEN DNase KIT. The RNA concentration and RNA integration number were assessed by NanoPhotometer and NanoDrop, respectively. The RNA samples with RIN > 7 were used to isolate mRNA from total RNA by the Dynabeads mRNA DIRECT Kit (Invitrogen) followed by library construction. Un-stranded specific RNA sequencing libraries were sequenced on the Illumina HiSeq 2500 (2 × 125 bp). Library construction and sequencing were commercially performed by Berry Genomics, Beijing, China. Obtained sequences were trimmed for the sequencing adaptors and for low-quality reads (*N* > 10% in a read) by Trimmomatic (Bolger et al., 2014). The sequence data quality of each sample was controlled by FastQC (Andrews, 2010).

### Transcriptome Profiling and Differentially Expressed Genes (DEGs) Detection

All trimmed transcriptome data were aligned to the chicken reference genome (GRCg6a) and annotation file (Gallus.gallus.GRCg6a.95.gtf) by the STAR software (version 2.5.3) (Dobin et al., 2013). Data were assembled by the StringTie software (version 1.3.3b) (Pertea et al., 2015). Gene- and transcript-level raw counts were calculated using the StringTie provided Python script with the parameter *l* = 125. The accuracy of the assembled files was evaluated by gffcompare (version 0.10.1) and included both coding and non-coding genes. Gene expression level normalization was performed by DESeq2 (Love et al., 2014), which is based on the experimental design as Stage + Tissue + Family. The normalized gene expression data were used for downstream analysis. The within-tissue PCA plots were performed by the distance of the samples calculated by rlog, and the PCA for all samples was performed by the distance of the samples calculated by the vst function of DESeq2 (Love et al., 2014). Using the Benjamini–Hochberg method (Benjamini and Hochberg, 1995) with adjusted *P* < 0.05, genes with expression fold change (FC) >1.5 or FC < 0.67 were considered as DEGs, which is based on the experimental design as Stage.

### Pathway Analysis

The KEGG enrichment and GO enrichment were performed by clusterProfiler package version 3.11.1 (Yu et al., 2012) with org.Gg.eg.db package version 3.8.2 (Carlson, 2019) and KOBAS 3.0 (Chen et al., 2011).

### Statistical Analysis

The Student *t*-test was performed using basic R (version 3.6.0) after using the function of the shapiro.test (for normality test) and the bartlett.test (for homogeneity test of variance). The data sets, which do not fit the normal distribution, were compared by the rank sum test (Kassambara, 2017). An LSD test was performed by the agricolae package (version 1.3.1) (De Mendiburu, 2014). Significance was stated at *P* < 0.05.

### Developmentally Dynamic Genes (DDGs) Identification in Two Tissues

The normalized gene expression data for the different developmental stages of BM and AF were used in the DDG analysis. Genes with average raw counts lower than 1 were excluded. The DDGs were identified by the maSigPro package (Ana et al., 2006; Nueda et al., 2014). By considering the expression distribution as the negative binomial model and the Benjamini–Hochberg procedure to adjust the FDR, significant genes were selected with the forward method using *r*^2^ > 0.7. Tissue pairwise comparison using gene expression patterns was performed with the same parameters described above. The list of TFs and TF cofactors (TFCFs) was acquired from AnimalTFDB (v.3.0) (Hu et al., 2018).

### Weighted Gene Co-expression Network Analysis (WGCNA)

All samples were used in the WGCNA except three BM outlier samples (Supplementary Figure S1). The remaining BM samples for the WGCNA did cover all the stages. A weighted gene co-expression analysis was performed by the WGCNA package (Langfelder and Horvath, 2008) with default settings and minor modifications. The minModuleSize was set to 100, and mergeCutHeight was set to 0.3 for tissue-stage-specific module detection (soft threshold = 9). A tissue-stage matrix for each RNA-Seq sample was built for correlation to identify the modules. For the WGCNA within-tissue data set, suitable soft-threshold power values were chosen based on the approximate scale-free topology for each analysis (soft threshold = 18 for BM in the E12 to D21 data set, soft threshold = 10 for BM in the D07 to D180 data set, and soft threshold = 5 for AF data set). By using the step-by-step topology overlap matrix (TOM), module detection, and similar module merging functions (minModuleSize = 30), gene module co-expression clustering dendrograms were built (Langfelder and Horvath, 2008). The module–traits associations were quantified, and the corresponding correlations were adjusted by the method of Benjamini–Hochberg. A *P*-value less than 0.01 of interesting module–traits’ corresponding correlations was used for further analysis. To identify the hub genes in the interesting modules, a customized hub gene filtering method was used. The gene network of each module was filtered as follows: (a) the edges with weight lower than 0.15 were removed; (b) the nodes with a connectivity number lower than 10 were removed; (c) the nodes with an average expression below 10 were removed, for controlling the false-positive rate; (d) finally, the genes were ranked by the summation of weight value. Gene co-expression networks were performed by Cytoscape software (version 3.7.0) (Shannon et al., 2003) with the edges provided by the WGCNA “exportNetworkToCytoscape” function.

## Results

### Phenotype Results: BM-IMF Percentage and AFW

**FIGURE 1:**
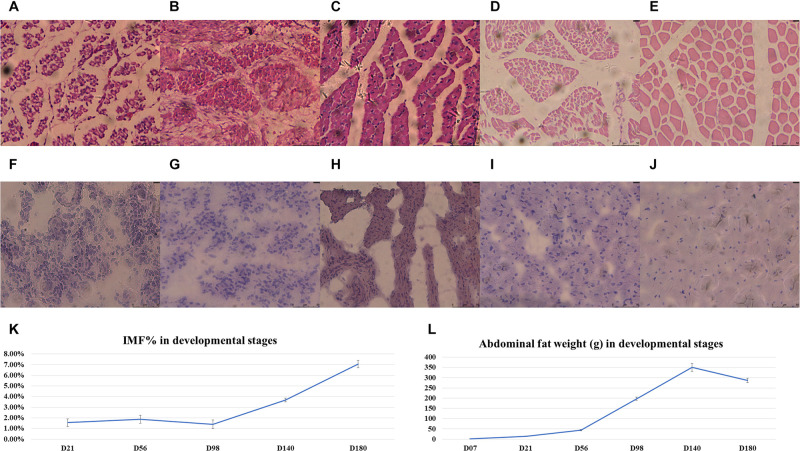
The phenotypes of the chickens used for RNA-Seq. **(A–E)** The HE-stained breast muscle sections in E12, E17, D01, D07, and D21. **(F–J)** The Red Oil O-stained breast muscle sections in E12, E17, D01, D07, and D21. The **(K)** IMF percentages and **(L)** average abdominal fat weight (gram); error bars are the standard deviations.

For the RNA-sequenced chickens, the IMF ratio of BM and the AFW at nine different stages was determined (Supplementary Table S1). The fitted growth curves of BM and AF for each of the three full-sib families during development are shown in Supplementary Figure S2, and both the BMW and AFW follow a logistic regression (*R*^2^ > 0.99). The HE-stained and Red Oil O-stained BM sections of E12, E17, D01, D07, and D21 are shown in Figures 1A–J, respectively. The HE-stained section of BM from E12 to D21 showed that the diameter of muscle fiber increased according to the developmental stage (Figures 1A–E). On the day of hatch (D01), the IMF% of BM was obviously high and then dropped to a low level at D07. From D21 to D98, IMF ratios stayed relatively constant, after which it gradually increased to 7.04% at D140 (Figure 1K). The AFW weekly gains from D56 to D98 and from D98 to D140 were significantly higher than those between other developmental stages, whereas D140 to D180 showed a decrease in AFW weekly gain (Figure 1L).

### Transcriptome Profiling

To obtain insight into the transcription of genes during the nine different developmental stages, transcriptome data were obtained from BM and AF from three individuals per stage. In total, 45 RNA-Seq libraries were constructed and sequenced (Table 1). After trimming of adaptors and removal of low-quality reads, an average of 28.58 million reads per library were aligned to the chicken reference genome (GRCg6a) with a mean alignment ratio of 92.48% over all libraries (Supplementary Table S2). In total, 21,853 genes were detected among all samples with 20,891 genes expressed in BM and 20,719 genes expressed in AF across all the tested developmental stages. We observed that 90.41% (19,757/21,853) of the genes were expressed in both BM and AF. The overlap of the BM and AF expressed genes is shown in Supplementary Figure S3. The gene raw read counts in each library are shown in Supplementary Table S3. The most highly expressed genes in BM and AF are *ACTA1* (actin alpha 1) and *MT-CO1* (cytochrome c oxidase subunit 1), respectively.

**FIGURE 2:**
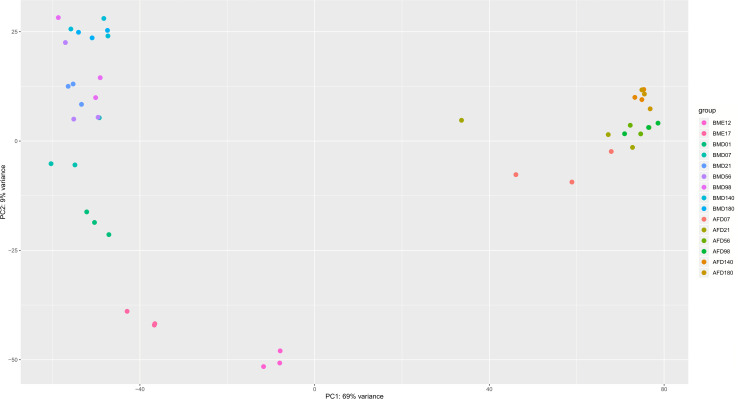
PCA plot of BM and AF samples at different developmental stages. The legends represent tissue plus developmental stage. BM stands for breast muscle, and AF stands for abdominal fat. The PCA was calculated by the vst function of the DESeq2 package based on the normalized raw gene counts.

To explore whether the expression profiles correlate with the developmental stages, a combined PCA of BM and AF expressed genes was performed (Figure 2) as well as individual PCA for BM and AF (Supplementary Figures S4A,B, respectively). As expected, there is a strong separation of the two tissues (Figure 2), whereas limited separations were observed for the developmental stages in the two tissues.

The stage-specific expressed gene numbers varied from 55 to 708 for BM and from 80 to 694 for AF (Supplementary Tables S4, S5 and Supplementary Figure S5A). The KEGG enriched pathways of the genes specifically expressed in different developmental stages are shown in Supplementary Figures S5B,C; e.g., the genes specially expressed in D01 were enriched for fatty acid-related pathways, such as fatty acid elongation, biosynthesis of unsaturated fatty acids, and fatty acid metabolism. The number of DEGs detected between the adjacent developmental stages varied from 13 to 1,432 for BM and from 48 to 1,177 for AF (Supplementary Table S6). The number of DEGs between the early stages is higher than that between the later stages of development. The number of DEGs between D140 and D180 in both BM and AF is relatively low. The KEGG enrichment pathways of DEGs between adjacent stages in BM and AF are shown in Supplementary Figures S6A,B. In the early stages of both BM and AF, the DEGs are enriched in the cell cycle and cell adhesion molecule pathways. The DEGs between D21 and D56 in BM are enriched in glycerolipid metabolism. The DEGs between D56 and D98 in AF are enriched in the steroid biosynthesis pathway.

### DDGs

**FIGURE 3:**
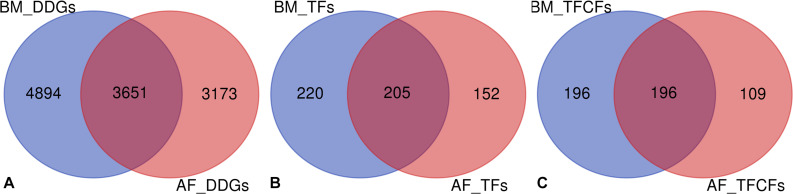
**(A)** Overlap of DDGs between BM and AF. **(B)** Overlap of DDG TFs between tissues. **(C)** Overlap of DDG TFCFs between BM and AF.

The genes which showed significant temporal changes in expression were identified in the two tissues, and these genes were considered as DDGs. The DDGs reflect the changes across developmental stages in gene expression regulation as well as in biological processes. In the BM data set, 8,545 genes were identified as DDGs, including 425 TFs and 392 TFCFs. In the AF data set, 6,824 DDGs were identified including 357 TFs and 305 TFCFs (Supplementary Table S7). On average, around one third of these DDGs overlap between the two tissues (Figures 3A–C). In contrast, the TFs and TFCFs of BM and AF DDGs are enriched in similar pathways, e.g., cellular senescence and AGE-RAGE signaling pathways in diabetic complications (Supplementary Figure S7). The full list of DDGs is presented in Supplementary Table S8. There are 37.48% and 31.48% of all currently identified TFs that are dynamically expressed in BM and AF, respectively.

### Expressed Genes in Different Developmental Stages

**FIGURE 4:**
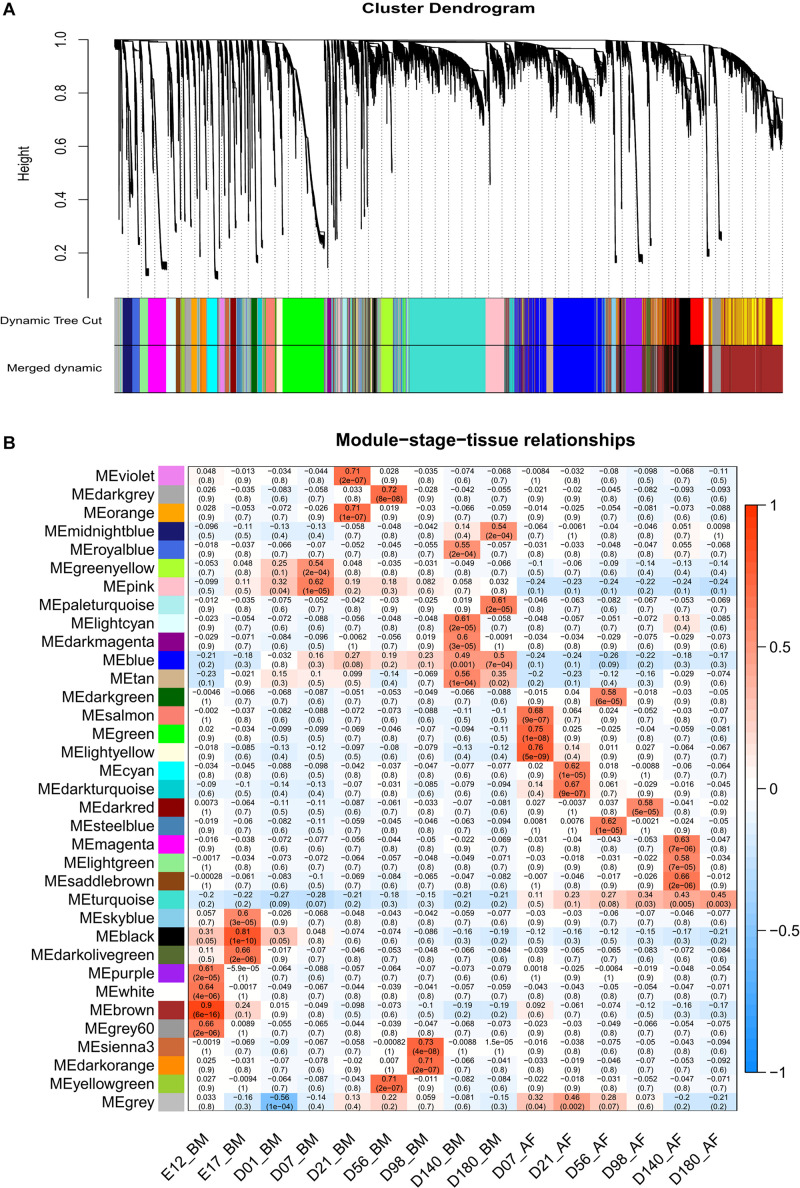
WGCNA results of BM and AF as a consensus data set. **(A)** Cluster dendrogram of the BM and AF. **(B)** Module–stage–tissue relationship of BM and AF.

From the whole data set, we investigated the stage-specific expressed genes in the BM and AF by WGCNA. The genes with similar expression patterns were clustered by the topology overlap matrix. The merged cluster dendrogram is shown in Figure 4A. In total, 34 co-expression gene modules were detected. A module can be considered as a group of clustered genes and is color-coded. The module–trait relationships of AF and BM are shown in Figure 4B, and the co-expressed gene modules were positively correlated with the developmental stages and the tissues. The genes in the cyan, light-yellow, black, and red modules are mainly expressed during the early stages of BM and are enriched for cell cycle, DNA replication, spliceosome, and mismatch repair pathways. The genes in the brown, white, purple, and grey60 modules expressed at E12 of BM are enriched for terms like cell cycle, spliceosome, RNA transport, DNA replication, mismatch repair, and homologous recombination.

### Detection of Hub Genes and Transcription (Co)factors Related to High IMF in BM

To detect the hub genes and the related transcription (co)factors involved in BM-IMF deposition, we performed the WGCNA on two different BM data sets. The rationale for two separate analyses is that BM has two adipocyte development phases. Thus, a WGCNA on the whole BM data set could result in potential false results. The first phase is from developmental stages E12 to D21 and is mainly related to adipocyte hyperplasia, and the second phase is from D07 to D180 mainly related to adipocyte hypertrophy. Consequently, developmental stages D07 and D21 were included in both phases.

**FIGURE 5:**
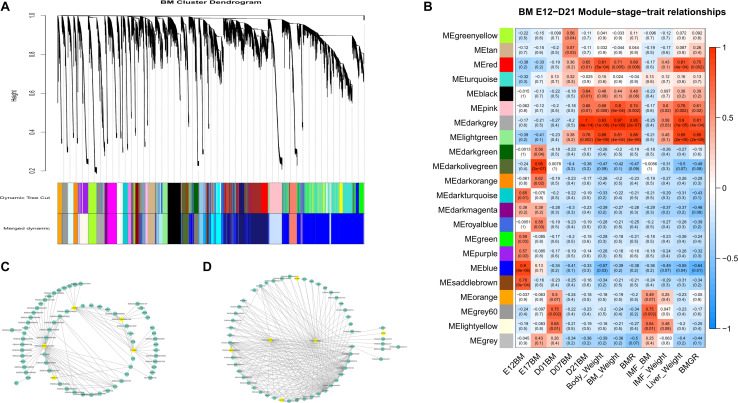
WGCNA results of the first-phase BM data set. **(A)** Module cluster dendrogram. **(B)** Module–trait relationship. The upper number in the block is the module’s corresponding correlation with the bottom trait, and the lower number is the *p*-value of the corresponding correlation. **(C)** Filtered co-expression network of the grey60 module. **(D)** Filtered co-expression network of the light-yellow module. The yellow nodes in **(C,D)** are the identified hub genes and involved TFs and TFCFs.

The module cluster dendrogram of the WGCNA results of the first phase in the BM data set (E12 to D21) is shown in Figure 5A, and the module–trait relationship is shown in Figure 5B. There are 22 modules clustered in the first-phase BM gene co-expression data set (Figure 5A). And the grey60 and light-yellow modules were significantly positively correlated with BM-IMF content (*P* = 0.002 and *P* = 0.01, respectively; Figure 5B). The network of eigengenes for this data set is shown in Supplementary Figure S8A. The co-expression network of the grey60 module is shown in Figure 5C. After filtering the edges with weight, connectivity, and the filtered weight summary of each node, the genes *ENSGALG00000053368*, *COX6A1*, *ATG9B*, and *ENSGALG00000041996* were identified as the hub genes in the grey60 module (Table 2). *ENSGALG00000041996* contacted genes are enriched in, e.g., carbon metabolism, valine degradation, fatty acid metabolism, 2-oxocarboxylic acid metabolism, and fatty acid elongation pathways. In the light-yellow module, *LOC107050564* and *ENSGALG00000048510* were identified as hub genes (Figure 5D). The detected TFs *MYCN* and *HOXB1* and the TFCFs *RNF168* and *ENSGALG00000008349* are involved in the light-yellow module.

**TABLE 2:** Hub genes, TFs, and enriched pathways identified in each phenotype-related module of breast muscle.

Data set	Module	Hub genes	TFs*	KEGG pathway**
BM Phase 1	Grey60	*ATG9B, COX6A1, ENSGALG00000041996, ENSGALG00000053368*	*HOXB1*	Carbon metabolism, leucine and isoleucine degradation, TCA cycle, fatty acid metabolism, fatty acid elongation
	Light yellow	*LOC107050564, ENSGALG00000048510*	*MYCN*	–
BM Phase 2	brown	*GIPC2, MLF1, UBE2V2, ENSGALG00000015443, ENSGALG00000030350*	*MYOD1*	Protein processing in the endoplasmic reticulum
	Dark gray	*ENSGALG00000050515, JCHAIN, LOC112532140*	*EGR2, EGR3, IRF5, KLF4, L3MBTL1, LITAF, PLEK, SMAD7B*	Cytokine–cytokine receptor interaction, neuro active ligand–receptor interaction, C-type lectin receptor signaling pathway

*Detailed TFs and TFCFs and pathways are shown in Supplementary Table S10. **There is no enriched pathway in the light-yellow module in the BM phase 1 data set. The detailed pathway is shown in Supplementary Table S11.

**FIGURE 6:**
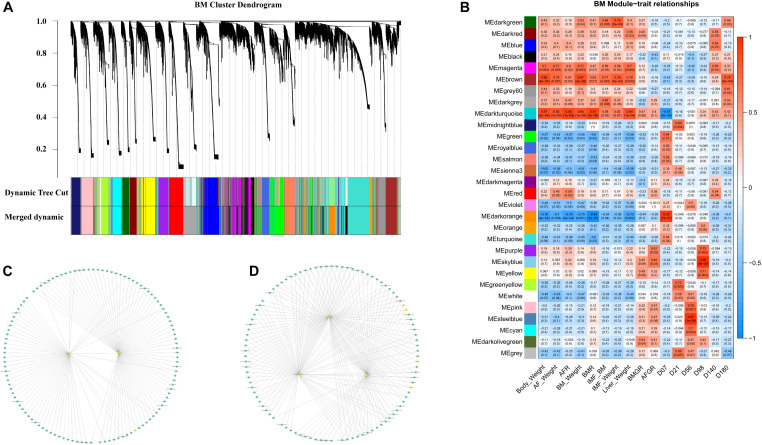
WGCNA results of the second-phase BM data set. **(A)** Module cluster dendrogram. **(B)** The module–trait relationship. **(C)** The filtered co-expression network of the brown module. **(D)** The filtered co-expression network of the dark-gray module.

Twenty-nine co-expression modules were detected for the second BM phase (D07–D180, Figure 6A), and the module–trait–stage relationships are shown in Figure 6B. The significant positive modules for BM-IMF percentage are the brown module (*P* = 0.003), the dark-green module (*P* = 0.005), and the dark-gray module (*P* = 0.008). The network of eigengenes for this data set is shown in Supplementary Figure S8B. After within-module edges filtering, no hub genes remained in the dark-green module. The genes *GIPC2* and *UBE2V2* in the brown module and *LOC112532140* and *ENSGALG00000053632* in the dark-green module were detected as hub genes related to the high BM-IMF percentage in phase 2 (Figures 6C,D). The involved TFs and gene enriched pathways are shown in Table 2.

### Detection of Hub Genes and Transcription (Co)factors Related to AFW

For the AF gene expression data in the WGCNA, the module cluster dendrogram (Supplementary Figure S9A) and the module–trait relationship (Supplementary Figure S9B) identified 24 modules. The network of eigengenes for AF expression data is shown in Supplementary Figure S9C. The turquoise module (*P* = 1e-06) is significantly positively related to AFW. After filtering the within-interaction edges, the genes such as *EIF3J*, *EPM2A*, *SH3BGRL*, *ENSGALG00000047756*, and *CHMP4B* were identified as hub genes. The turquoise module membership-vs.-gene significance on AFW is shown in Supplementary Figure S9D.

The yellow module, with 1,099 genes, is significantly negatively related to AFW (*P* = 0.009). The yellow module membership-vs.-gene significance for AFW is shown in Supplementary Figure S9E. Several hub genes were identified, such as *MASTL*, *CENPE*, *MCM4*, *CREB3L1*, and *PKB*. The genes of the solute carrier family, such as *SLC1A3*, *SLC2A10*, *SLC3A1*, *SLC6A8*, and *SLC7A2*, were also involved in the co-expression network. The complete list of involved TFs and TFCFs derived from the WGCNA is provided in Supplementary Tables S9, S10. The TFs involved and genes enriched in the pathways are shown in Table 3.

**TABLE 3:** Hub genes, TFs, and enriched pathways identified in AFW-related module of AF.

Module	Correlation	Hub genes	TFs*	KEGG pathway**
Turquoise	Positively correlated to AFW	*EIF3J, EPM2A, CALM1*	*PPARD, CEBPB, CEBPD, EGR2, EGR4, FOS, MXD4, RREB1, TWIST2, XBP1*	Endocytosis, lysosome, mRNA surveillance pathway, and proteasome
Yellow	Negatively correlated to AFW	*MASTL, CENPE, MCM4, CREB3L1*	*FOXM1, CREB3L1, MYBL2, GATA4, TULP3, RFX5, L3MBTL1*	Cell cycle, protein processing in the endoplasmic reticulum

*Detailed TFs and TFCFs and pathways are shown in Supplementary Table S10. **The detailed KEGG enriched pathway is shown in Supplementary Table S11.

The TF *L3MBTL1* and the TFCFs *TNIP1*, *HAT1*, and *BEND6* are both involved in the IMF positively related module of the second phase of the BM data set and overlapped in the AFW negatively related module of the AF data set.

## Discussion

Time course RNA sequencing has been widely used to study cellular differentiation (Ma et al., 2018), tissue development (Cardoso-Moreira et al., 2019), and aging (Baumgart et al., 2016). We provide a new insight on the transcriptome changes in chickens between different development stages of BM and AF. Although the Jingxing-Huang IMF-up selected chicken population is an inbred line, the experimental chickens have similar genetic background. The three biological replicates cannot cover all the population transcriptome changes but provide an indication of the function involved in fat metabolism in chicken BM. The PCA result indicates that the transcriptome changes of the late developmental stages are smaller than those of the early stages. The numbers of stage-specific expressed genes of early stages are higher than those of later stages. The IMF ratio of BM displays a peak around the day of hatch followed by an increase from D98 to D180, which is in agreement with an earlier study focusing on the stages around the day of hatch (Liu et al., 2017). On the day of hatch (D01), the IMF content is high (13.6%), compared to embryotic stages and D07 to D98. This might relate to the elevated BM growth during this period. The genes specifically expressed in BM during stage D01 are enriched for fatty acid elongation and biosynthesis of unsaturated fatty acids, indicating that these pathways may contribute to the high IMF phenotype in BM. After hatch, most of the BM-IMF deposition starts around D98, while the AF deposition starts from D07 and accelerates from D56.

Genes with significant changes in expression at different developmental stages were considered as DDGs. We used DDGs to reflect the transcriptome of BM and AF changes during development in cell type abundance, gene regulation, and the proportion of cells undergoing division (Pantalacci and Sémon, 2015). The number of DDGs in BM (8,545) and AF (6,824) is somewhat higher than the average number of DDGs detected in Red jungle fowl (RJF) in the brain, cerebrum, heart, kidney, liver, ovary, and testis (Cardoso-Moreira et al., 2019). There are several possible explanations for the observed differences, e.g., the tissues, sequencing technology, time points, and species. The number of DDGs in BM is higher than that in AF, showing that compared with AF tissue, BM tissue has more genes that change in expression during the developmental period assessed in this study. This could be due to the higher number of cell types in BM compared to AF. Furthermore, the number of TFs and TFCFs decreased during development, which is consistent with earlier research in other animals (Bolger et al., 2014). Thus, as the development process proceeds, the required number of TFs becomes lower.

WGCNA is a powerful tool for identifying genes that are associated with the phenotypes under study (Langfelder and Horvath, 2008). WGCNA can also be used to identify tissue- or stage-specifically expressed gene modules (Gao et al., 2018; Ma et al., 2018). To investigate the expressed genes in different developmental stages, we performed the WGCNA for BM and AF tissues as a consensus data set. Thirty-four modules were detected, indicating that the gene expression varies a lot between the developmental stages. There are 2,157 genes in the salmon, green, and light-yellow modules with more than 39% genes with unknown function. The genes in the turquoise module are expressed higher in AF than in BM and are enriched in the *PPAR* signaling and fatty acid metabolism pathways, which are known to be involved in fat deposition.

We initially performed the WGCNA on the complete BM data set. We found that IMF positively related modules are similar to the positively related D01 stage modules, representing the IMF deposition during the early period. This may be an issue to cause potential false-positive errors in the early stages of adipocyte differentiation as well as false-negative errors in the late stage for fat deposition. Therefore, two separate WGCNAs were performed for the hyperplasia and the hypertrophy phases of the BM data. The hyperplasia phase is covered by E12 to D21, and the hypertrophy phase includes stages D07 to D180. For the AF data set, there are only samples from D07 to D180. Hence, there is no divided phase in the AF data set. There are different ways to identify the hub genes for WGCNA results; e.g., the WGCNA package provides a function for hub gene detection (Langfelder and Horvath, 2008), and the genes with kME >0.95 can also be considered as hub genes (Gao et al., 2018). However, in this study, there are several large modules, which may be driven by several hub genes. Then, we used the expression level of genes, the weight of connected genes, and the connectivity number of genes as the criteria for the detection of hub genes.

In the WGCNA results of the first-phase BM data set, the hub gene *ENSGALG00000041996*, an lncRNA, may regulate *CD36* and *ACADL*. The *ENSGALG00000041996* connected genes in BM phase 1 data set enriched pathways are shown in Supplementary Figure S10. This would make sense because fatty acids are transported via fatty acid binding protein (FABP), fatty acid translocase (FAT/CD36), and cell membrane diffusion (Stump et al., 1993). The acyl-CoA dehydrogenase long-chain gene (*ACADL*) plays a role in catalyzing the first step of mitochondrial fatty acid beta-oxidation (Indo et al., 1992). Both the *CD36* and *ACADL* belong to the *PPAR* signaling pathway. In contrast, the *PPAR* signaling pathway can also induce and activate the expression of *aP2* and *PEPCK*, which are specifically expressed in fat tissue (Tontonoz et al., 1995). This suggests that the unannotated gene *ENSGALG00000041996* may play a key role in fat deposition during the early developmental stages of BM. In the second-phase BM data set WGCNA, the brown module is significantly positively correlated with the BM-IMF. *MYOD1* is the only TF in the brown module. *MYOD1* is also connected with the hub gene *GIPC2* (Figure 6C). This may indicate that the TF *MYOD1* regulates genes in the brown module through *GIPC2*, thereby affecting muscle development and IMF deposition. For the other hub genes, especially the genes with very limited knowledge about function, such as *ENSGAL0000005538*, *LOC107050546*, *ENGSGALG00000015443*, and *ENSGALG00000030350*, they may play yet undescribed regulatory roles in IMF deposition. As the phenotypes of BMW and IMF have very similar patterns, we cannot distinguish if the genes are associated with muscle development or IMF deposition. The identified hub genes and involved TFs in the BM data set can be used as candidate genes for high-IMF chicken selection.

To identify the hub genes involved in the AF deposition, we performed the WGCNA in the AF data set. There is no obvious relationship between the identified hub genes and the lipid metabolism, e.g., *EIF3J* (eukaryotic translation initiation factor 3 subunit J), *EPM2A* (epilepsy, progressive myoclonus type 2A), and *CALM1* (calmodulin 1). However, the TFs *PPARD* and *CEBPB* are involved in the turquoise module, which is positively correlated to AFW. The gene *PPARD* is expressed in multiple tissues in adult mouse (Higashiyama et al., 2007) and regulates glucose metabolism and insulin sensitivity (Chih-Hao et al., 2006). *CEBPB* seems to be synergistic in promoting lipogenesis in AF of cockerels (Resnyk et al., 2017). The TF *CREBP3L1* (cAMP responsive element binding protein 3 like 1) was involved in the AFW negatively correlated yellow module, while the gene *CREBP* can reduce the lipogenesis as well as glycolysis in mice (Katsumi et al., 2004). The solute carrier family genes, which are involved in the yellow module, did not show in the center of the yellow module. This indicates that the genes of the solute carrier family may play some roles in the downstream of lipid metabolism.

In chicken breeding, there is a desire of producing chickens with high IMF and low AF. From our study, we found some promising candidate genes. Particularly, the TF *L3MBTL1* and the TFCFs *TNIP1*, *HAT1*, and *BEND6*, which were identified as significantly positively related to the high IMF and significantly negatively related to the low AFW, could be relevant biomarkers for chicken breeding. RT-qPCR on the four TFs/TFCFs in BM of Jingxing-Huang and Cobb chickens in a large number of individuals has recently been investigated (Li et al., 2020). It was shown that the expressions of *TNIP1* and *HAT1* in the high-IMF group are significantly higher than those in the low-IMF group, supporting the role of at least these two genes in the fat metabolism of chickens (Li et al., 2020).

## Conclusion

In this manuscript, the transcriptome dynamics of chicken BM and AF in different developmental stages are described. This is an important resource for studying IMF and AF in chicken. Developmental dynamics genes and involved TFs were identified, which may play key roles in tissue development. In addition, we identified several regulatory hub genes that potentially can be used in breeding to improve IMF content in muscle while reducing the AFW.

## Data Availability Statement

The datasets analyzed for this study can be found in the Genome Sequence Archive [53] in BIG Data Center [54], Beijing Institute of Genomics (BIG), Chinese Academy of Sciences, under project number PRJCA001192 and accession number CRA001334 that are publicly accessible at http://bigd.big.ac.cn/gsa.

## Ethics Statement

The animal experiments were conducted at the chicken farm in the IAS of CAAS, and the animal experiments had been approved by the animal ethics committee of Institute of Animal Sciences, Chinese Academy of Agricultural Sciences in 2016 (approved code IASCAAS-AE-02).

## Author Contributions

JW, GZ, RL, RC, MG, and SX designed the study. SX, LL, and MZ performed the animal experiments and sample collection. LL, SX, and XY tested the phenotypes. SX performed the data analysis and wrote the manuscript. RL, RC, OM, MG, and SX discussed the results. OM, RL, RC, and MG provided valuable suggestion and comments to improve the manuscript with contributions from all other authors. All authors contributed to the article and approved the submitted version.

## Conflict of Interest

The authors declare that the research was conducted in the absence of any commercial or financial relationships that could be construed as a potential conflict of interest.

## Footnotes

1 http://www.fao.org/faostat/en/#home

## Supplementary Material

FIGURE S1 The overlap between BM and AF expressed genes.

FIGURE S2 **(A)** The PCA plot of BM samples, **(B)** the PCA plot of AF samples.

FIGURE S3 **(A)** The stage specifically expressed gene number of BM and AF. **(B)** BM stage specifically expressed genes enriched KEGG pathways. **(C)** AF stage specifically expressed genes enriched KEGG pathways.

FIGURE S4 The BM IMF and AFW fitted curve.

FIGURE S5 The TFs and TF cofactors of BM and AF DDGs enriched pathways.

FIGURE S6 **(A)** BM samples outliers. **(B)** The network of eigengene for the WGCNA result of the first phase of the BM data set. **(C)** The network of eigengenes for the WGCNA result of the second phase of the BM data set.

FIGURE S7 **(A)** WGCNA result of modules dendrogram. **(B)** module traits relationship. **(C)** AFW eigengene dendrogram and adjacency heatmap. **(D)** Turquoise module membership vs. gene significance on AFW. **(E)** Yellow module membership vs. gene significance on AFW.

FIGURE S8 The KEGG pathway of *ENSGALG00000041996* related genes in the grey60 module.

TABLE S1 Phenotypes of RNA-sequenced chickens.

TABLE S2 Alignment summary for each sequenced sample.

TABLE S3 Gene expression raw counts matrix. Library names were developmental stage plus tissue plus family ID.

TABLE S4 BM stage specifically expressed gene list.

TABLE S5 AF stage specifically expressed gene list.

TABLE S6 The number of DEGs between each developmental stage in BM and AF. The number of up- and down-regulated gene number between adjacent developmental stages in each tissue from D07 to D180. The comparison performed as former stage vs. latter stage.

TABLE S7 BM and AF expressed TFs of DDGs number in different stages.

TABLE S8 Developmental dynamic gene list of BM and AF.

TABLE S9 Transcription factors list of DDGs and interested modules.

TABLE S10 Transcription factors co-factors of DDGs and interested modules.

TABLE S11 Details of KEGG enriched pathways of interested module of WGCNA results.

## Abbreviations

*ACADL*: acyl-CoA dehydrogenase long chain
AF: abdominal fat
AFGR: abdominal fat growth rate
AFW: abdominal fat weight
BM: breast muscle
BMGR: breast muscle growth rate
*CREBP3L1*: cAMP responsive element binding protein 3 like 1
DDGs: developmental dynamic genes
DEGs: differentially expressed genes
IMF: intramuscular fat
MSCs: mesenchymal stem cells
*MYOD1*: myogenic differentiation 1
*PPAR* γ: peroxisome proliferator activated receptor γ
RJF: red jungle fowl
TF: transcription factors
TOM: topological overlap matrix
WGCNA: weighted gene co-expression network analysis.
