# Supplementary figures and images for: RNA-Seq Analysis Reveals Hub Genes Involved in Chicken Intramuscular Fat and Abdominal Fat Deposition During Development
Source: Front Genet. 2020 Aug 28;11:1009. doi: 10.3389/fgene.2020.01009 (PMC7493673; doi:10.3389/fgene.2020.01009)

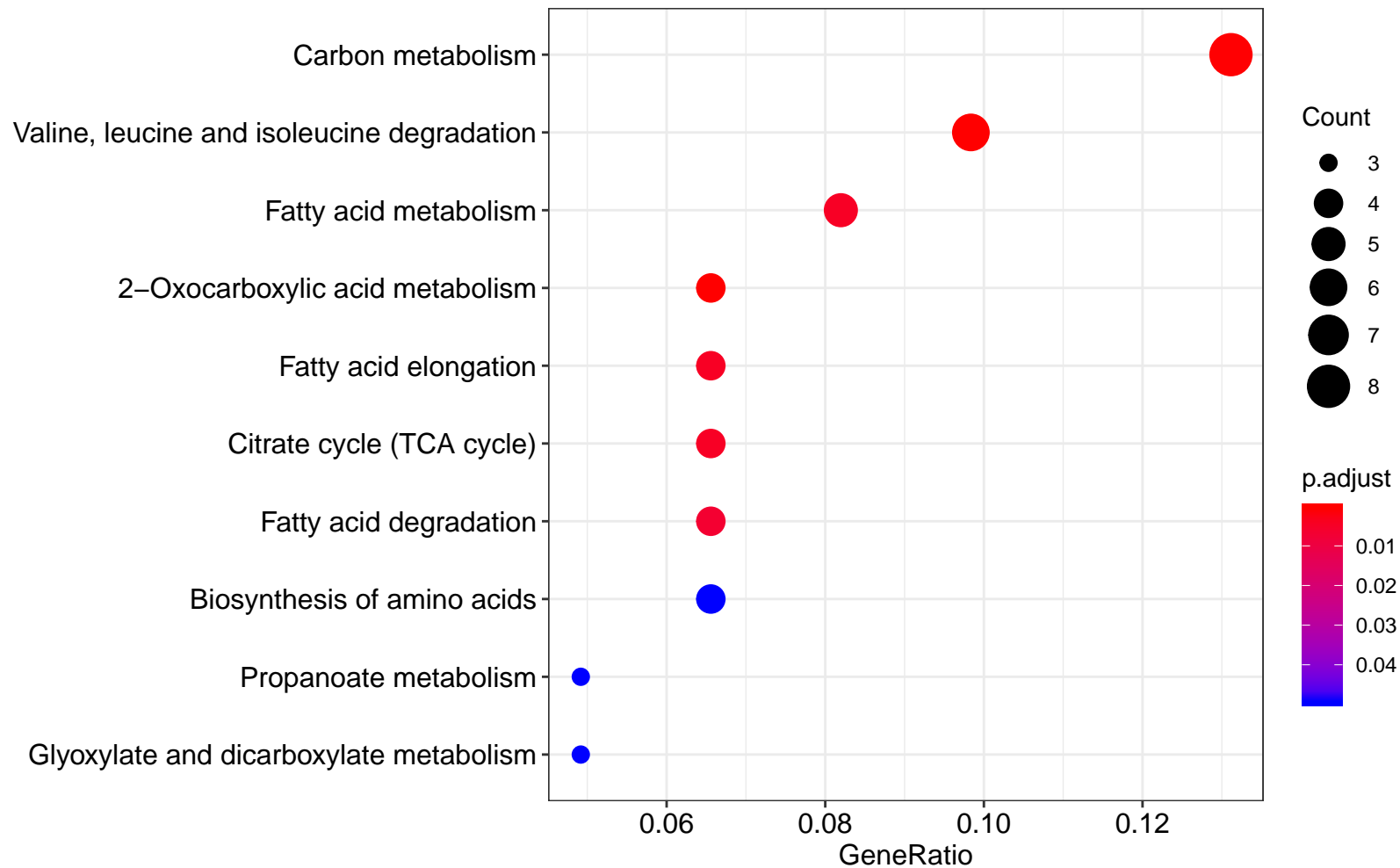

Supplement: Supplementary file 2 [file Image_10.PDF]

Sample clustering to detect outliers\_BM

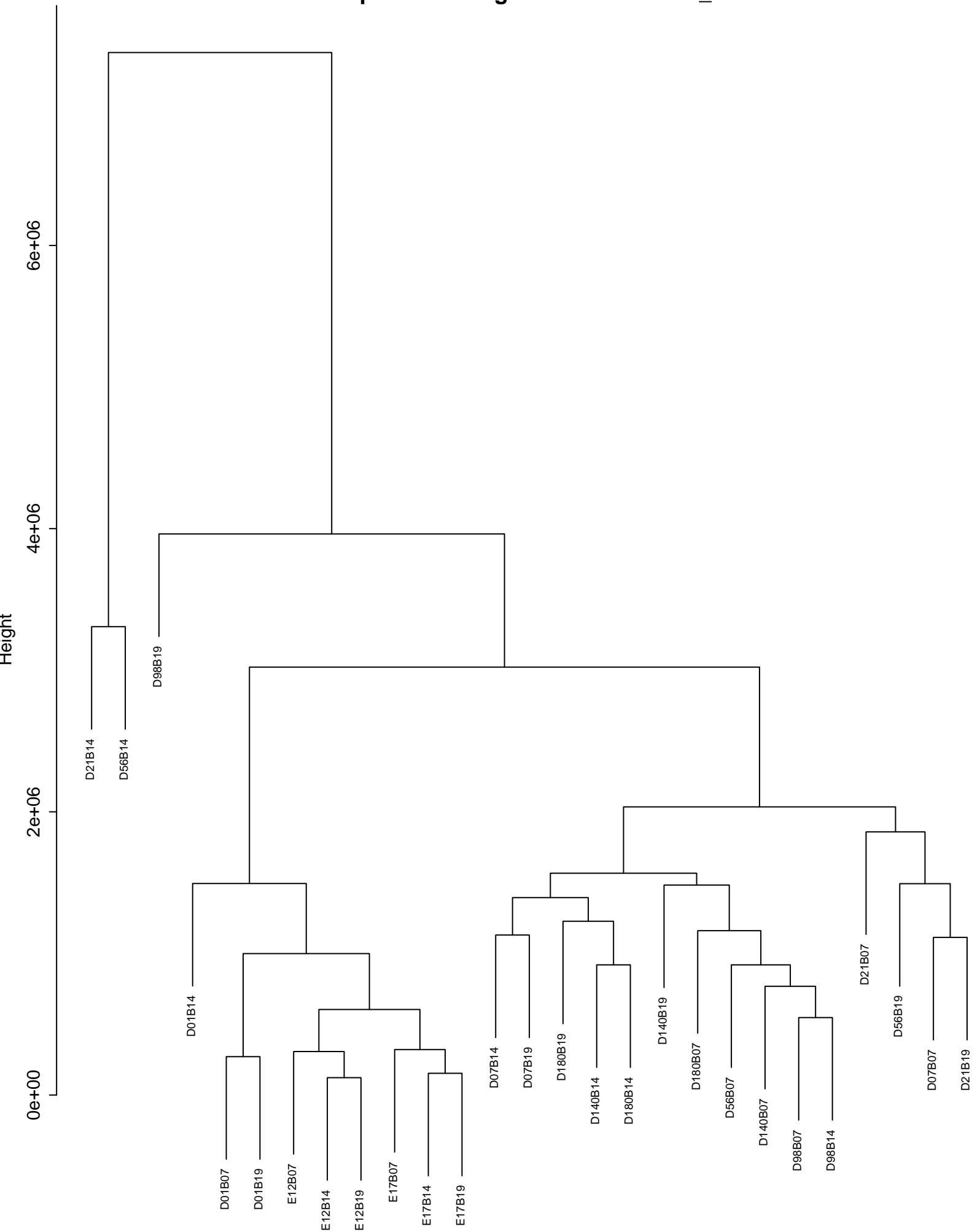

Supplement: FIGURE S1 — The overlap between BM and AF expressed genes. [file Image_1.PDF]

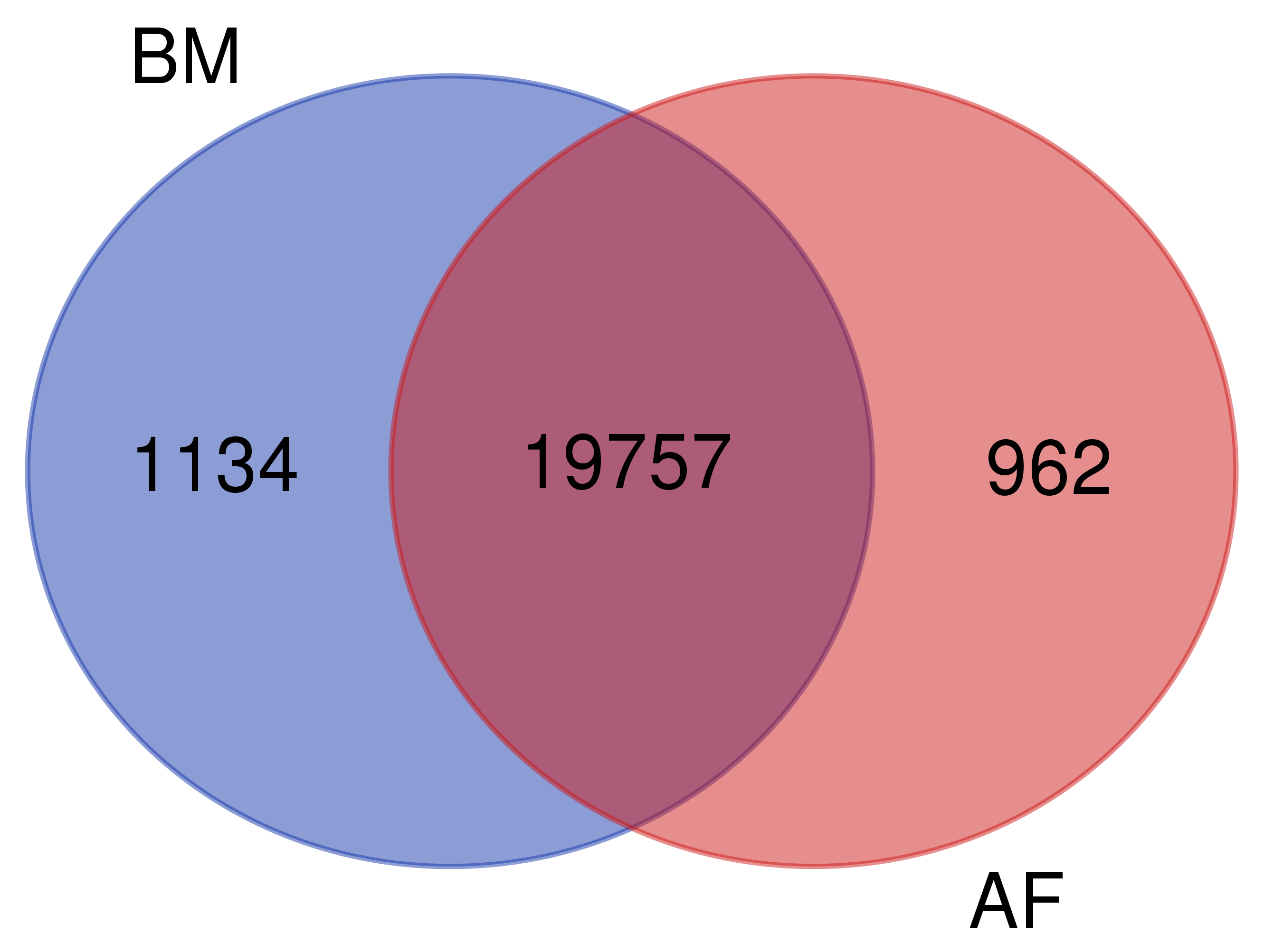

Supplement: FIGURE S3 — (A) The stage specifically expressed gene number of BM and AF. (B) BM stage specifically expressed genes enriched KEGG pathways. (C) AF stage specifically expressed genes enriched KEGG pathways. [file Image_3.PNG]

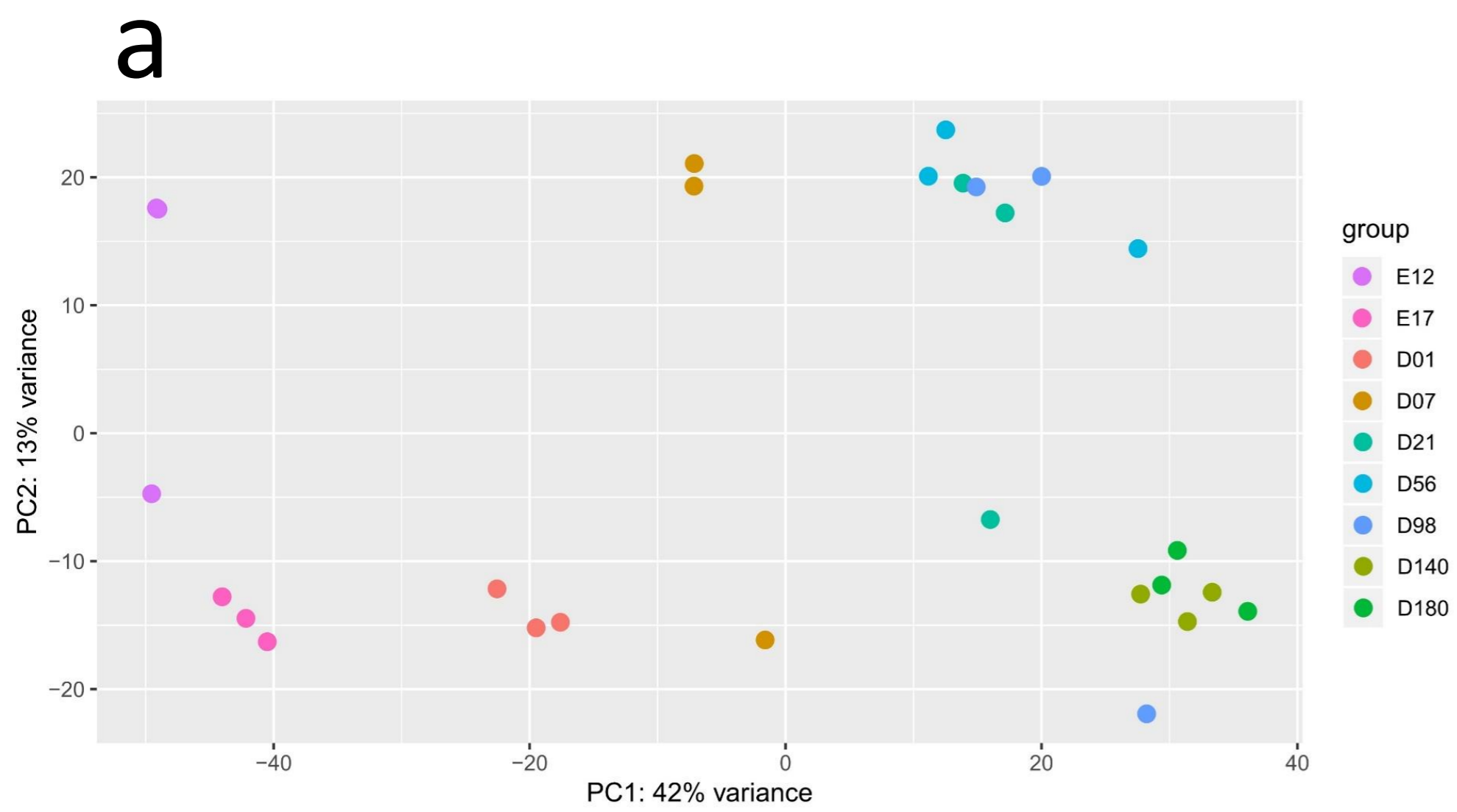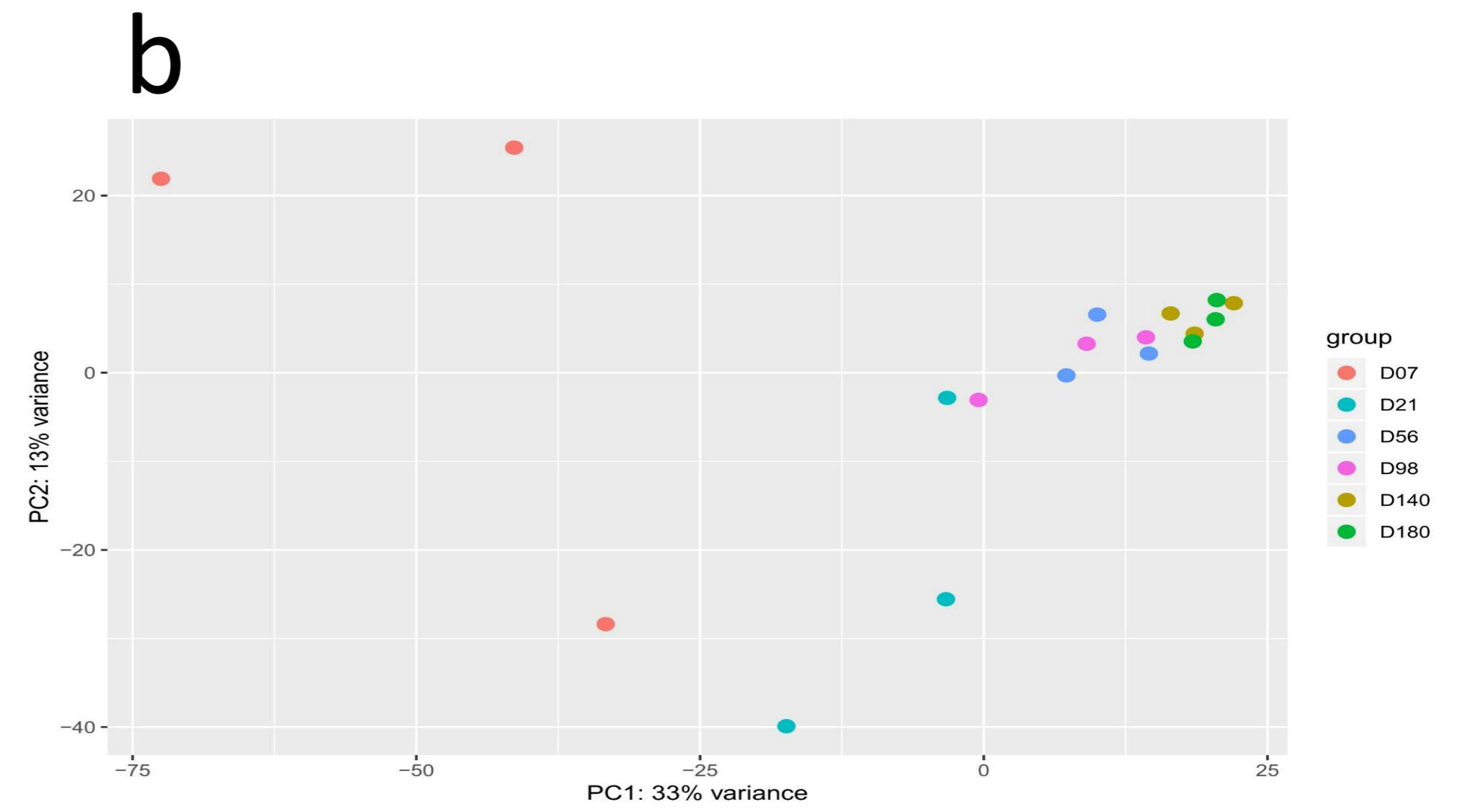

Supplement: FIGURE S4 — The BM IMF and AFW fitted curve. [file Image_4.PDF]

Stage specifically expressed gene number

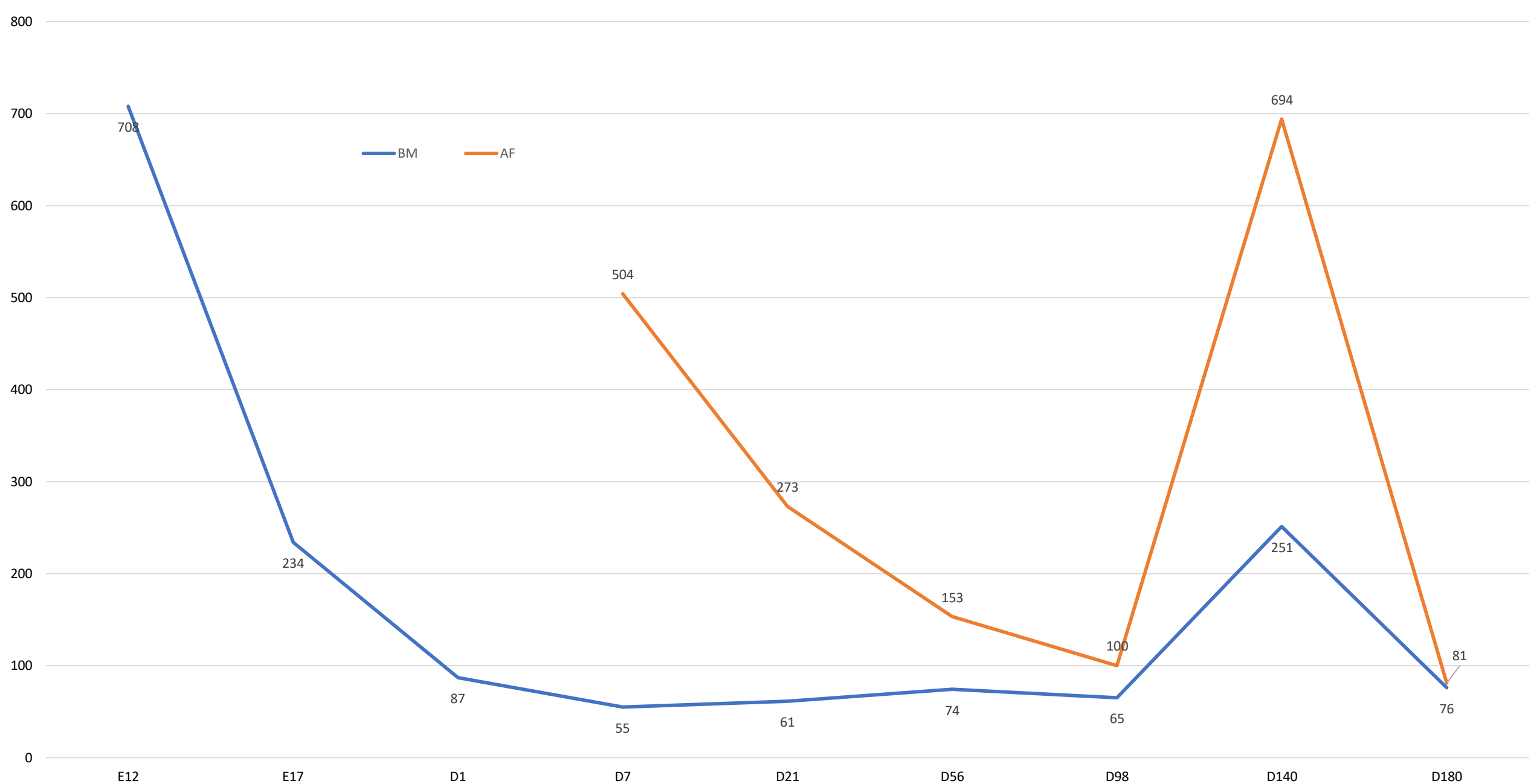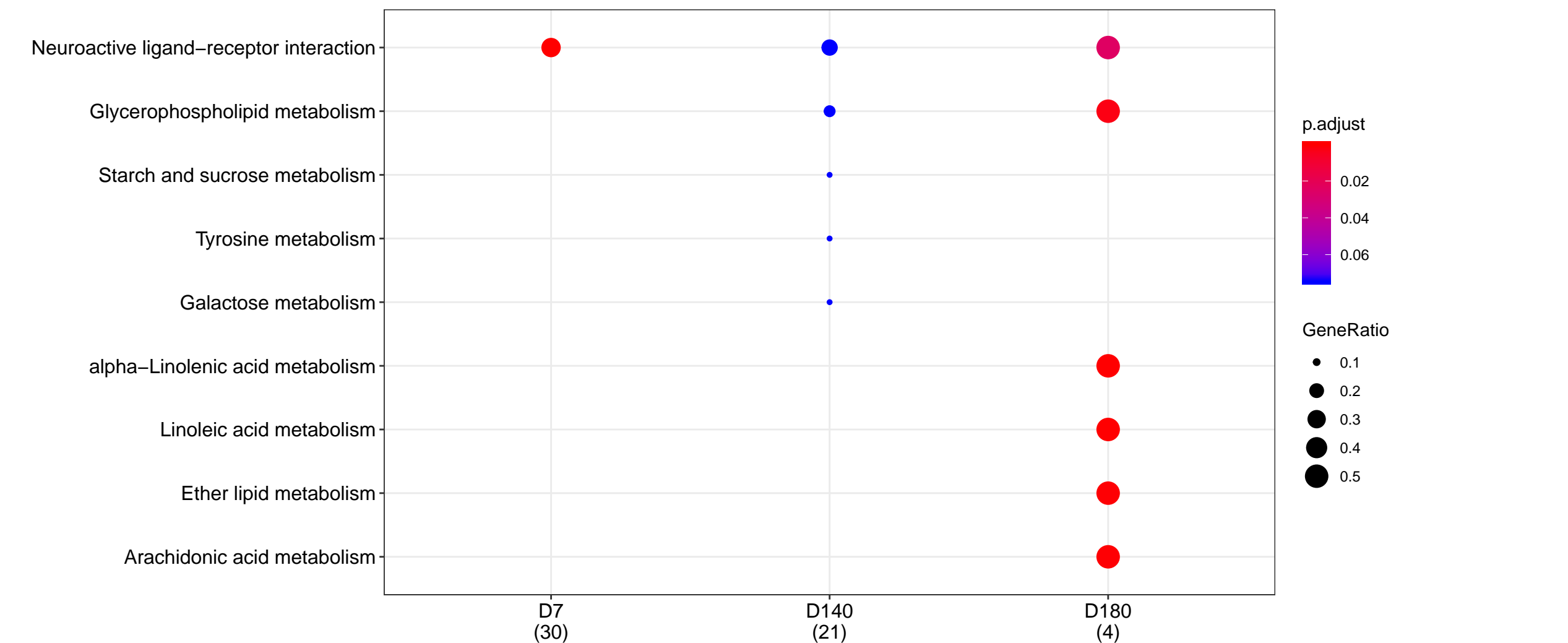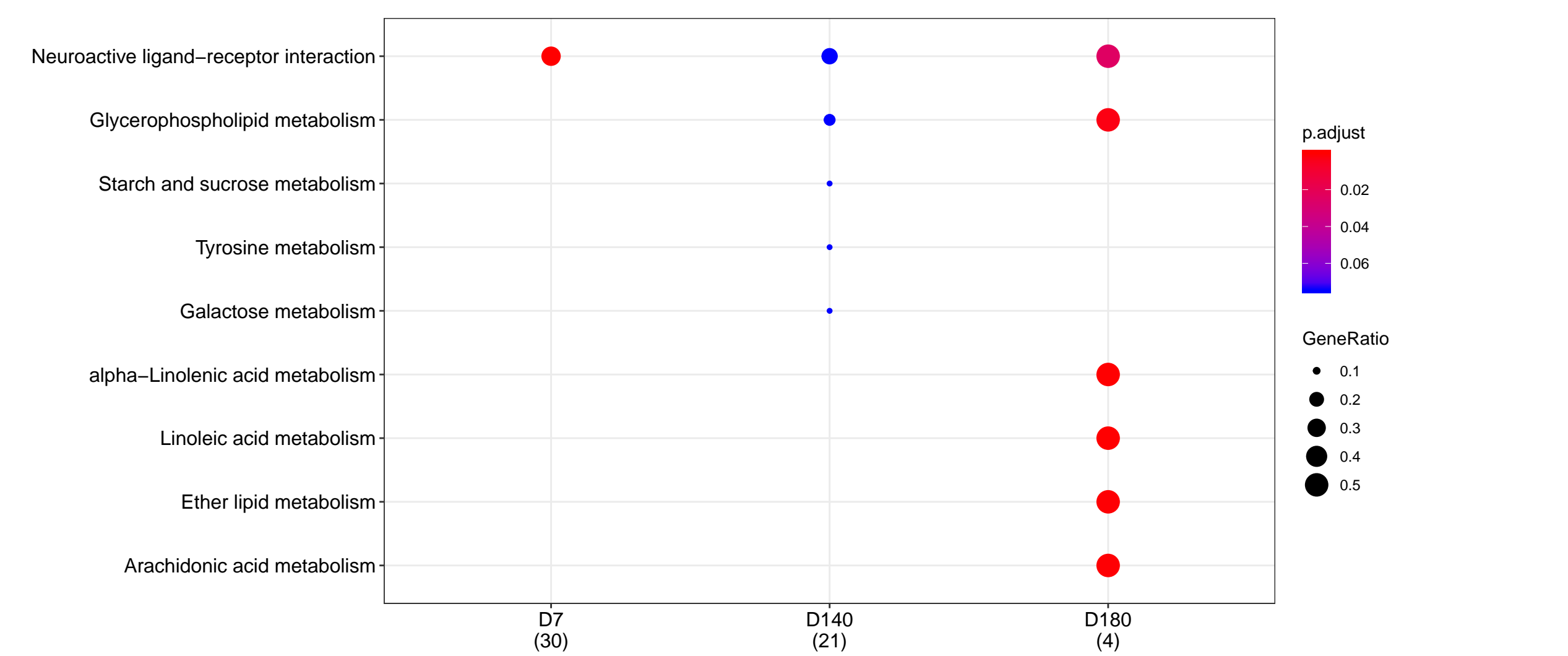

Supplement: FIGURE S5 — The TFs and TF cofactors of BM and AF DDGs enriched pathways. [file Image_5.PDF]

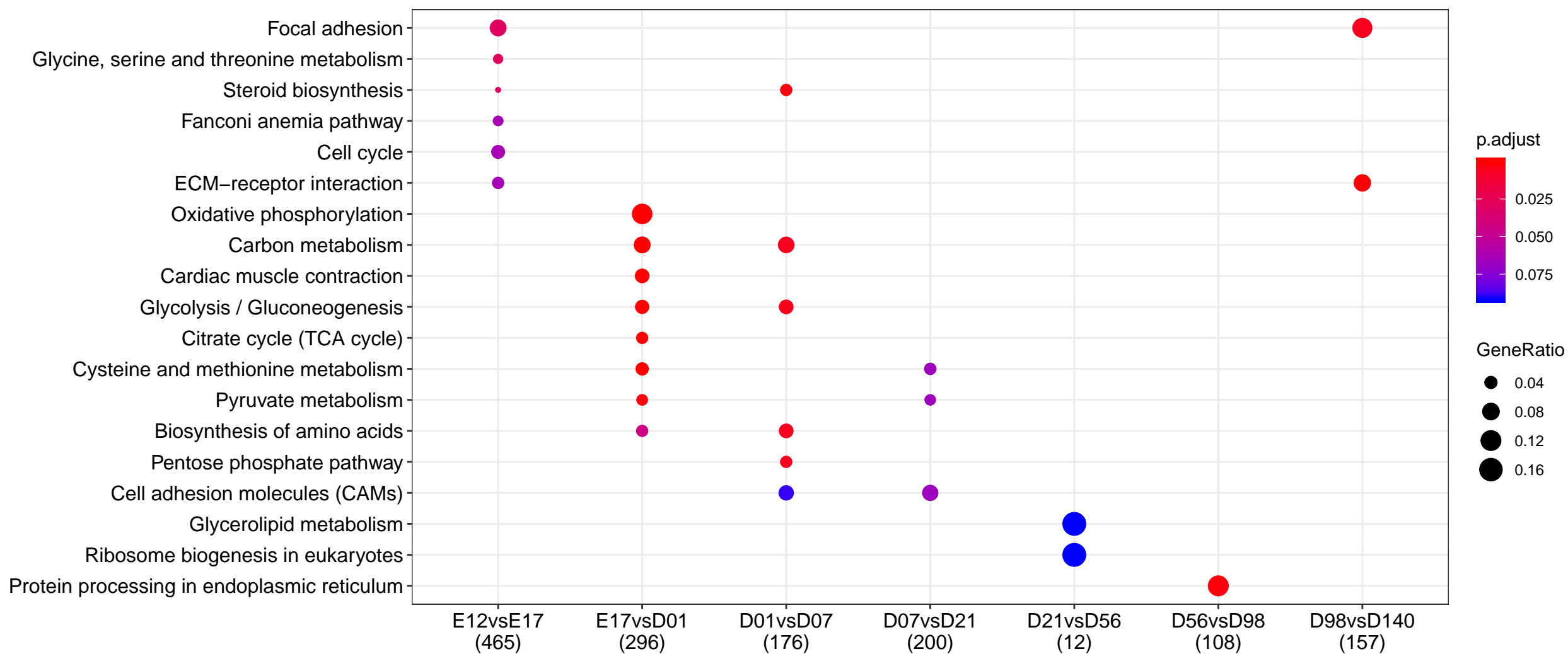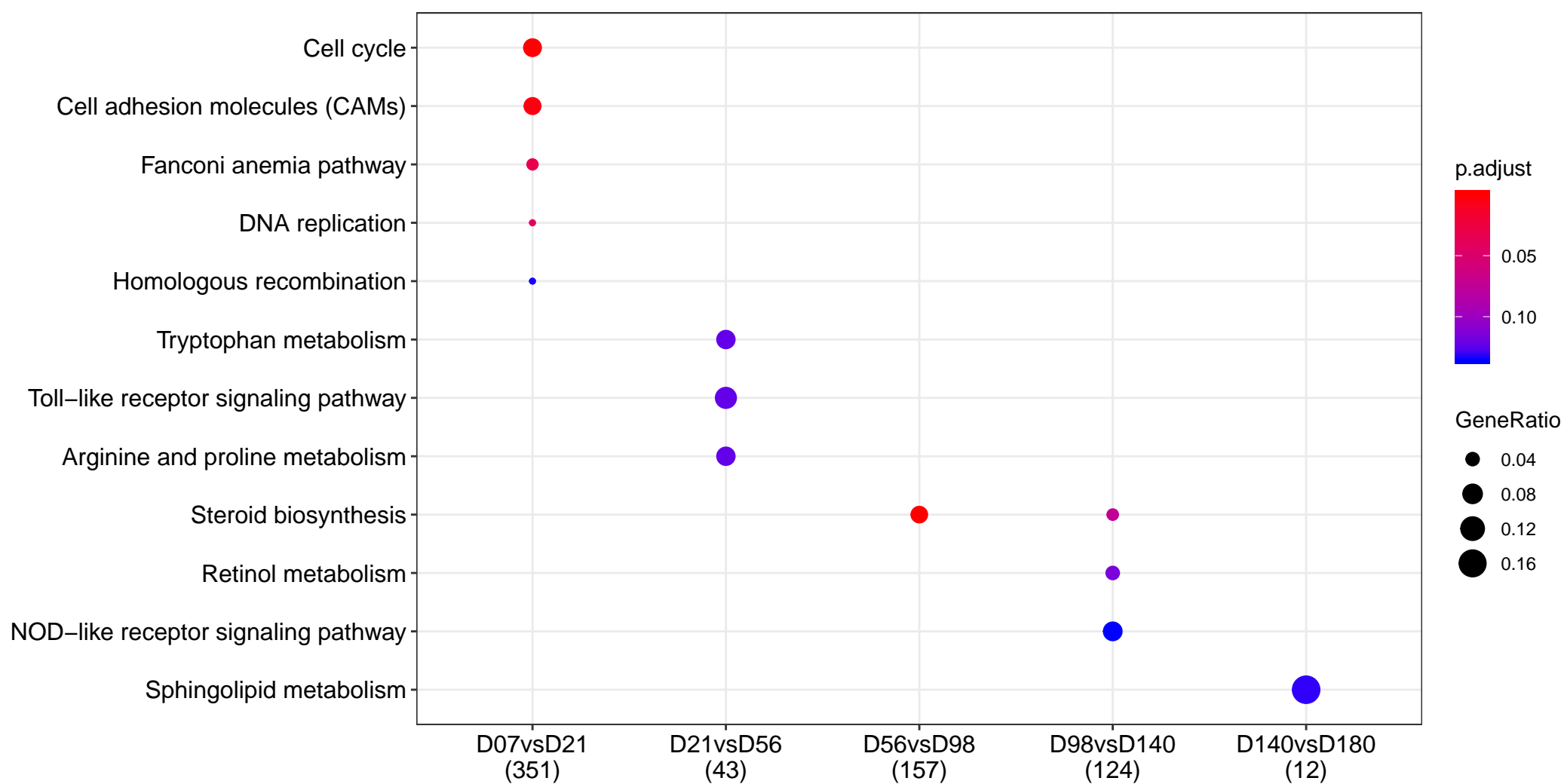

Supplement: FIGURE S6 — (A) BM samples outliers. (B) The network of eigengene for the WGCNA result of the first phase of the BM data set. (C) The network of eigengenes for the WGCNA result of the second phase of the BM data set. [file Image_6.PDF]

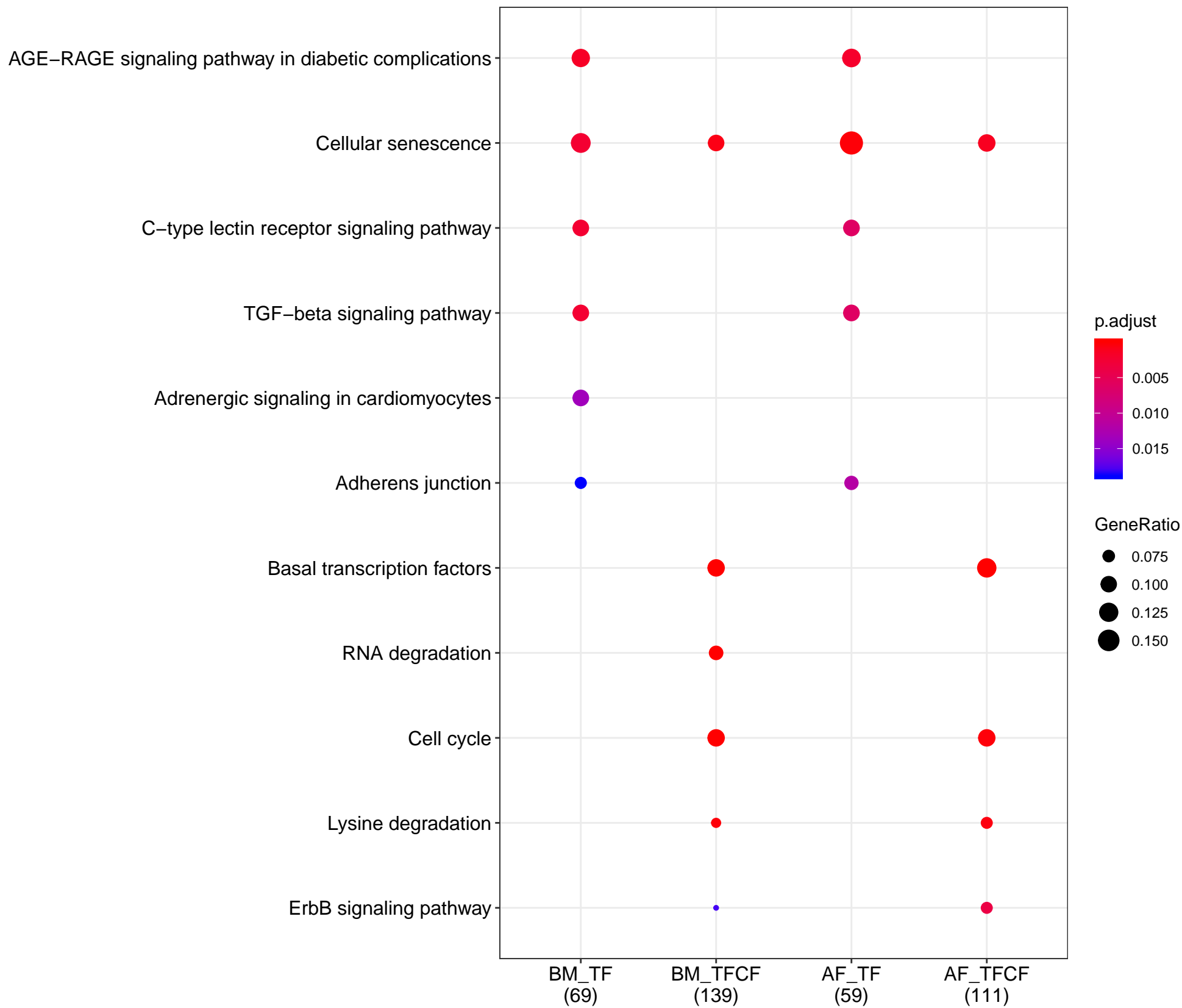

Supplement: FIGURE S7 — (A) WGCNA result of modules dendrogram. (B) module traits relationship. (C) AFW eigengene dendrogram and adjacency heatmap. (D) Turquoise module membership vs. gene significance on AFW. (E) Yellow module membership vs. gene significance on AFW. [file Image_7.PDF]

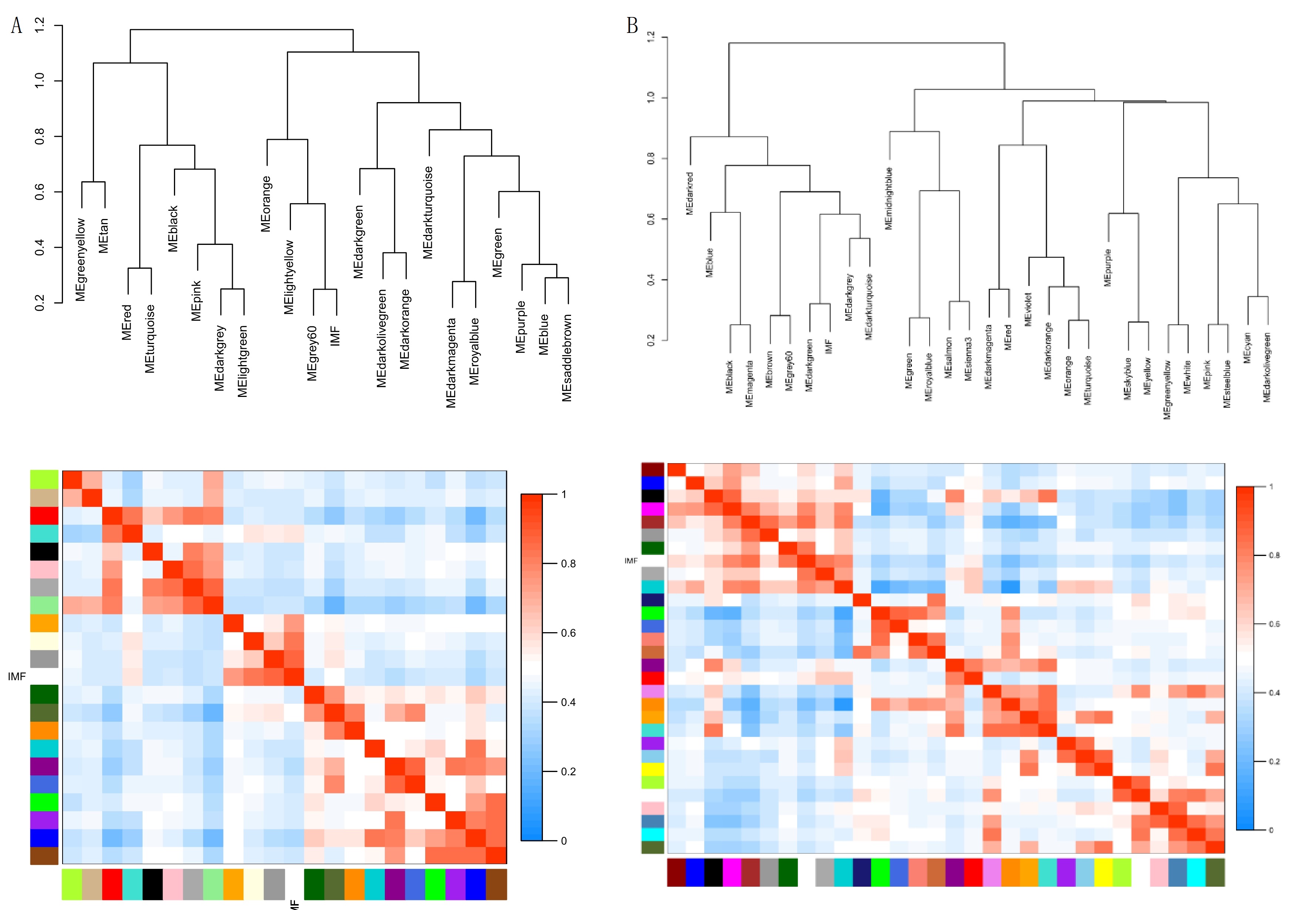

Supplement: FIGURE S8 — The KEGG pathway of ENSGALG00000041996 related genes in the grey60 module. [file Image_8.JPEG]
