# Supplementary material for: RNA-Seq Analysis Reveals Hub Genes Involved in Chicken Intramuscular Fat and Abdominal Fat Deposition During Development
Source: Front Genet. 2020 Aug 28;11:1009. doi: 10.3389/fgene.2020.01009 (PMC7493673; doi:10.3389/fgene.2020.01009)
Supplement: FIGURE S2 — (A) The PCA plot of BM samples, (B) the PCA plot of AF samples. [file Image_2.PDF]

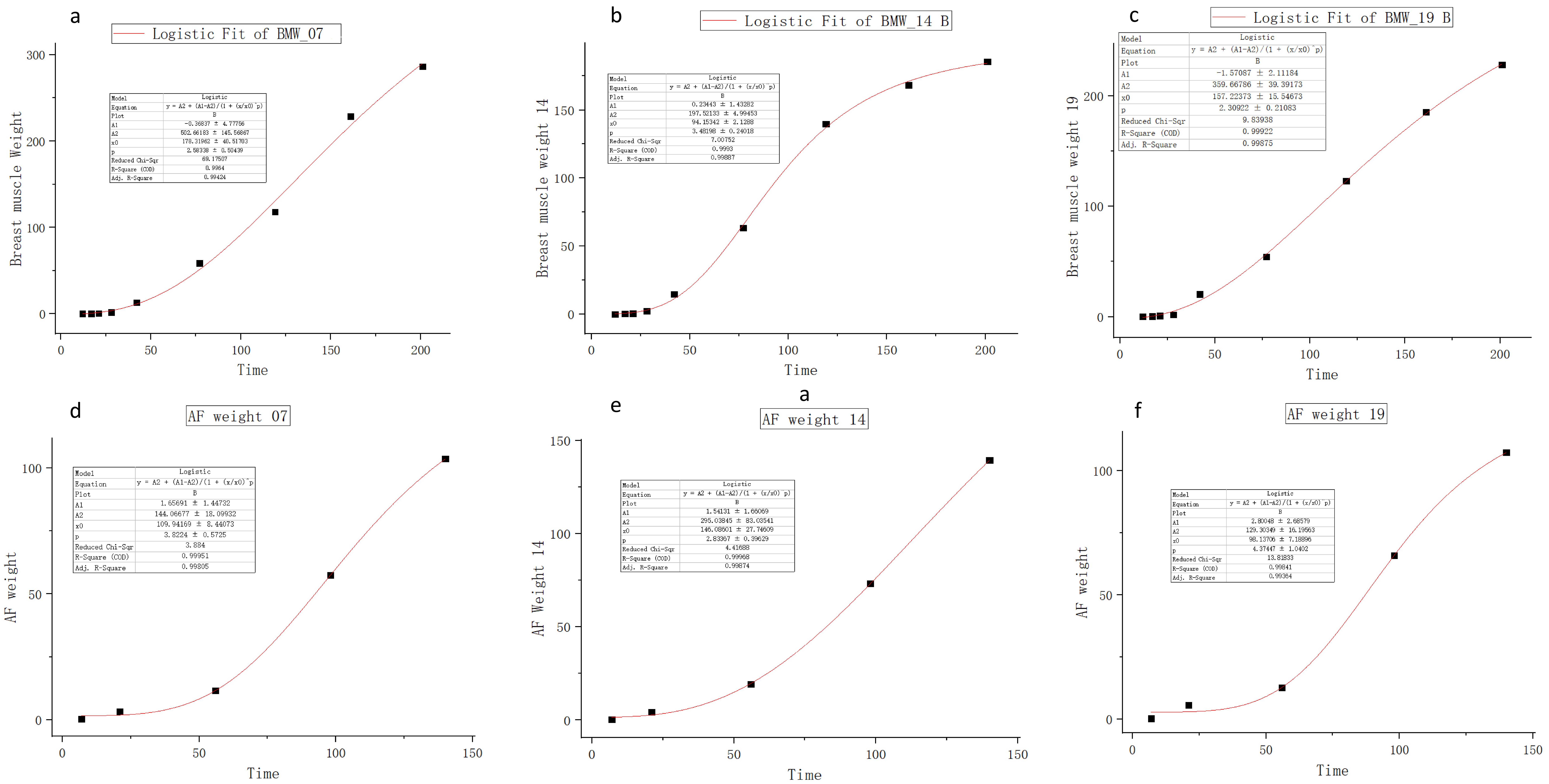
a
Logistic Fit of BMW_07
Breast muscle Weight
Time
0
50
100
150
200
300
Model | Logistic
Equation | y = A2 + (A1-A2)/(1 + (x/x0)^p)
Plot | B
A1 | -0.36837 ± 4.77756
A2 | 502.66183 ± 145.56867
x0 | 178.31962 ± 40.51703
p | 2.58338 ± 0.50439
Reduced Chi-Sqr | 69.17507
R-Square (COD) | 0.9964
Adj. R-Square | 0.99424
b
Logistic Fit of BMW_14 B
Breast muscle weight 14
Time
Model | Logistic
Equation | y = A2 + (A1-A2)/(1 + (x/x0)^p)
Plot | B
A1 | 0.23443 ± 1.43282
A2 | 197.52133 ± 4.99453
x0 | 94.15342 ± 2.1288
p | 3.48198 ± 0.24018
Reduced Chi-Sqr | 7.00752
R-Square (COD) | 0.9993
Adj. R-Square | 0.99887
c
Logistic Fit of BMW_19 B
Breast muscle weight 19
Time
Model | Logistic
Equation | y = A2 + (A1-A2)/(1 + (x/x0)^p)
Plot | B
A1 | -1.57087 ± 2.11184
A2 | 359.66786 ± 39.39173
x0 | 157.22373 ± 15.54673
p | 2.30922 ± 0.21083
Reduced Chi-Sqr | 9.83938
R-Square (COD) | 0.99922
Adj. R-Square | 0.99875
d
AF weight 07
AF weight
Time
Model | Logistic
Equation | y = A2 + (A1-A2)/(1 + (x/x0)^p
Plot | B
A1 | 1.65691 ± 1.44732
A2 | 144.06677 ± 18.09932
x0 | 109.94169 ± 8.44073
p | 3.8224 ± 0.5725
Reduced Chi-Sqr | 3.884
R-Square (COD) | 0.99951
Adj. R-Square | 0.99805
e
a
AF weight 14
AF Weight 14
Time
Model | Logistic
Equation | y = A2 + (A1-A2)/(1 + (x/x0)^p)
Plot | B
A1 | 1.54131 ± 1.66069
A2 | 295.03845 ± 83.03541
x0 | 146.08601 ± 27.74609
p | 2.83367 ± 0.39629
Reduced Chi-Sqr | 4.41688
R-Square (COD) | 0.99968
Adj. R-Square | 0.99874
f
AF weight 19
AF weight
Time
Model | Logistic
Equation | y = A2 + (A1-A2)/(1 + (x/x0)^p)
Plot | B
A1 | 2.80048 ± 2.68579
A2 | 129.30349 ± 16.19563
x0 | 98.13706 ± 7.18896
p | 4.37447 ± 1.0402
Reduced Chi-Sqr | 13.81833
R-Square (COD) | 0.99841
Adj. R-Square | 0.99364
